# A Bibliometric Analysis of the Oligometastatic State over the Last Two Decades: A Shifting Paradigm for Oncology? An AIRO Oligometastatic Study Group

**DOI:** 10.3390/cancers15153902

**Published:** 2023-07-31

**Authors:** Giulia Marvaso, Federico Mastroleo, Giulia Corrao, Mattia Zaffaroni, Maria Giulia Vincini, Paolo Borghetti, Francesco Cuccia, Manuela Federico, Giampaolo Montesi, Antonio Pontoriero, Davide Franceschini, Ciro Franzese, Marta Scorsetti, Barbara Alicja Jereczek-Fossa

**Affiliations:** 1 Division of Radiation Oncology, European Institute of Oncology IRCCS (IEO), 20141 Milan, Italy; giulia.marvaso@ieo.it (G.M.); federico.mastroleo@ieo.it (F.M.); giulia.corrao@ieo.it (G.C.); mattia.zaffaroni@ieo.it (M.Z.); barbara.jereczek@ieo.it (B.A.J.-F.); 2 Department of Translational Medicine, University of Piemonte Orientale (UPO), 13100 Novara, Italy; 3 Radiation Oncology Department, ASST Spedali Civili and University of Brescia, 25123 Brescia, Italy; paolo.borghetti@asst-spedalicivili.it; 4 Radiotherapy Unit, ARNAS Civico Hospital, 90127 Palermo, Italy; francesco.cuccia@sacrocuore.it; 5 Casa di cura Macchiarella, U.O. Radioterapia Oncologica, 90127 Palermo, Italy; manuela.fed@gmail.com; 6 Radiation Oncology Department, ‘S.M. Della Misericordia’ Hospital, AULSS 5 Veneto, 45100 Rovigo, Italy; giampaolomontesi@gmail.com; 7 Department of Biomedical, Dental and Morphological and Functional Imaging Sciences, University of Messina, 98122 Messina, Italy; antonio.pontoriero@unime.it; 8 Radiation Oncology Unit, A.O.U. Policlinico “G. Martino” Messina, 98124 Messina, Italy; 9 Department of Radiotherapy and Radiosurgery, IRCCS Humanitas Research Hospital, Via Manzoni 56, 20089 Milan, Italy; davide.franceschini@unimed.eu (D.F.); ciro.franzese@hunimed.eu (C.F.); marta.scorsetti@hunimed.eu (M.S.); 10 Department of Biomedical Sciences, Humanitas University, 20090 Pieve Emanuele, Italy; 11 Department of Oncology and Hemato-Oncology, University of Milan, 20122 Milan, Italy

**Keywords:** radiotherapy, oligometastatic state, bibliometrics, oncology

## Abstract

**Simple Summary:**

The concept of “oligometastatic state” has brought about a shift in our understanding of cancer’s metastasis and progression, and its management remains an active field of research, with ongoing efforts to develop new treatment options. The aim of the present study is to report a bibliometric analysis of the oligometastatic disease/state to provide an overview of the topic. A total of 3304 documents were collected from 1995 to 2022. Among all keywords used by authors, the top three were oligometastases (19%), SBRT (18%), and radiation therapy (8%). In particular, a link can be observed between radiotherapy and topics of side-fields like immunotherapy and targeted therapies, representing an opportunity for further combinatorial studies in this setting. This bibliometric analysis represents a detailed summary of the most influential studies dealing with the oligometastatic state and sheds light on the area for the attention of researchers worldwide.

**Abstract:**

The term “oligometastasis” represents a relatively novel idea, which denotes a condition characterized by cancer dissemination with a limited number of lesions (usually fewer than five). The aim of the present study is to report a bibliometric analysis of the oligometastatic disease/state, incorporating all relevant studies on the topic for more than 20 years. The research strategy included at least one the terms “Oligmetastases”, “Oligometastasis”, “Oligometastatic”, “Oligoprogression, “Oligoprogressive”, “Oligorecurrent”, or “Oligorecurrency” in the title, abstract, and/or keywords. All English-language documents from 1 January 1995 (the year of the earliest available document in Scopus) to 31 December 2022 were considered for the analysis. R code (R version 4.2.0) with R Studio (version 2022.12.0-353) and the Bibliometrix package (version 4.0.1) were used for the analysis. A total of 3304 documents, mainly articles (*n* = 2083, 63.0%) and reviews (*n* = 813, 24.6%), were collected from 1995 to 2022. The average annual growth rate of literature on the topic was 26.7%. Overall 15,176 authors published on the topic, with an average of eight authors/publication. From 1995, 69 countries contributed to the literature, with the USA and Italy being the top contributors. Among all keywords used by authors, the top three were oligometastases (19%), SBRT (18%), and radiation therapy (8%). Themes regarding “locoregional treatment”, “organ motion”, and “immunotherapy” were the most recent trend topics, mainly developed from 2019 to 2022, while “high-dose chemotherapy”, “whole-brain radiotherapy”, and “metastatic breast cancer” saw their main development during 2009–2018. Our study shows the exceptionally flourishing scientific production on the oligometastatic state, summarizing the most influential studies and highlighting the future developments and interests. This analysis will serve as a benchmark to identify this area for the attention of researchers worldwide and contribute to the increasing scientific work.

## 1. Introduction

The concept of “oligometastatic state” represents a relatively novel idea, which has brought about a shift in our understanding of cancer’s metastasis and progression in general. Historically, cancer metastasis was considered to be a late-stage event associated with poor prognosis. However, the understanding of oligometastasis has led to the realization that some patients with limited metastatic disease may have a better prognosis than previously thought [1,2]. Indeed, the term “oligometastasis” denotes a condition characterized by cancer’s dissemination to a limited number of body sites—usually fewer than five [2,3,4,5]. The oligometastatic condition is most commonly observed in patients with solid tumors, such as lung, prostate, and breast cancers [6,7,8,9,10].

The diagnosis of oligometastasis relies on a limited set of criteria: timing (synchronous vs. metachronous), location of metastases (bone vs. visceral), and number of localizations on imaging detection (traditional vs. advanced) [2]. The enhanced detection capability of relatively new imaging methodologies such as PSMA-PET/CT and whole-body MRI may allow for the early detection of small lesions that would have been imperceptible on conventional imaging [11]. Once oligometastasis is diagnosed, treatment options may include surgery, stereotactic body radiotherapy (SBRT), systemic therapy, or a combination of these treatments.

The management of oligometastasis remains an active field of research, with ongoing efforts to develop new treatment options. Over the past two decades, the oligometastatic state has garnered significant interest from the oncology community, particularly among radiation oncologists, sparking a growing interest in carving out a new role for local treatments and metastasis-directed therapy (MDT), such as SBRT [12,13,14,15,16,17].

Bibliometric analysis relies on the application of statistical methods to the study of the published literature. The field of bibliometrics encompasses the quantitative aspects of scientific communication, including the number of publications, citations, and authorship patterns. Bibliometric studies are used to gain insights into the productivity, influence, impact, and visibility of individual scholars, journals, and institutions.

The quantity of publications on a specific topic is among the most commonly used bibliometric indicators. In the present study, a bibliometric analysis of the current literature on oligometastatic disease/states was conducted, incorporating all relevant studies on the topic for more than 20 years, since the first publication of the definition proposed by R. Weichselbaum and S. Hellman in 1995 [18]. 

## 2. Materials and Methods

### 2.1. Data Origin and Search Strategy

The Scopus electronic documents database was used as a data source (consulted on 19 January 2023). The search strategy included at least one of the terms “Oligometastases”, “Oligometastasis”, “Oligometastatic”, “Oligoprogression, “Oligoprogressive”, “Oligorecurrent”, or “Oligorecurrency” in the title, abstract, and/or keywords. All English-language documents from 1 January 1995 (the year of the earliest available document in Scopus) to 31 December 2022 were deemed eligible for the analysis. Data were exported using the BibTeX file format. The BibTeX file format is comprehensive of all of the metadata regarding citation information (i.e., authors, document title, year, source title, volume, issue, pages, citation count, source and document type, publication stage, DOI, and open-access status), bibliographical information (i.e., PubMed ID, affiliations, publisher, editors, document language, correspondence address, abbreviated source title), abstract and keywords, and references included in the manuscript. 

### 2.2. Data Analysis

R code (R version 4.2.0) with R Studio (version 2022.12.0-353) and the Bibliometrix package (version 4.0.1 [19]) were employed to perform the present analysis. The “convert2df” function was used to obtain dataframes from the transformed BibTeX files, followed by the application of the “biblioAnalysis” command and “Summary()” function from the Bibliometrix package. This enabled an exploratory analysis to be carried out, highlighting key characteristics such as the total number of papers published per year, the growth rate, the top active countries and their related production as deduced from the first author’s affiliation, the most highly cited papers, and the most prominently displayed journals. Plots were created for the primary data. The “citations” command was used to analyze further references. The “metaTagExtraction” and “Biblionetwork” tools were used to analyze collaboration networks, and “Networkplot” was utilized for graphical depiction. The international cooperation ratio of a given country was calculated by considering documents in which at least one of the co-authors was from a different country than the corresponding author. The command “Biblioshiny()” was used to perform additional analyses through a visual user interface and to create maps for national scientific collaborations, institution collaboration networks, and a full co-occurrence network. Machine learning clustering technology was used to analyze keywords, and trend subjects were highlighted. Co-occurrence network analysis of authors’ keywords was performed using the walktrap clustering algorithm [20]. Thematic map analysis [21] was built on the clusters identified by machine learning, showing their degree of development (density) and relevance (centrality). This strategic diagram enabled the identification of hot topics (i.e., higher centrality and density values) in the upper-right quadrant, basic topics (i.e., higher centrality and lower density values) in the lower-right quadrant, peripheral topics (i.e., lower centrality and density values) in the lower-left quadrant, and niche topics (i.e., lower centrality and higher density values). A factorial technique was used to map the conceptual structure, with the goal of reducing the data’s dimensionality and displaying them in a low-dimensional environment. Close keywords indicate a large proportion of articles addressing them jointly, whereas distant keywords indicate just a tiny fraction of articles addressing them simultaneously. The average location of all column profiles indicated the center of the study field, comprising common and widely discussed themes. Each color in the correspondence analysis and clustering word map represents a cluster of words or a subject found by hierarchical clustering [22]. 

A linear regression analysis was performed to obtain a prevision of the expected article in 2023. Tidyverse and caret R libraries were used. The set.seed function was used to set a seed for the random number generator, ensuring that the results would be reproducible. The data were equally divided into a training set and a test set. The linear regression model was fitted to the training data using the lm function, with the number of articles as the response variable and the year as the predictor variable (with a polynomial function up to the fifth degree). Model performance was evaluated using the root-mean-square error (RMSE) and R-squared (R^2^) metrics. The results were plotted using the ggplot function, where a red point represents the year 2023 and its corresponding number of expected articles.

## 3. Results

### 3.1. Overview

**Figure 1:**
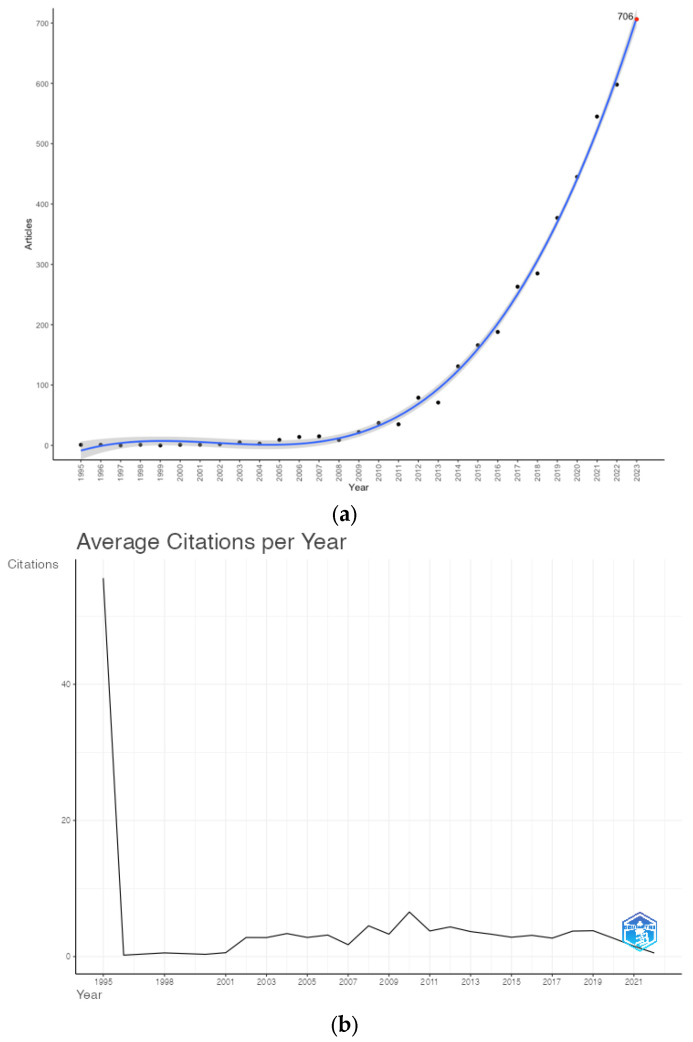
(**a**) Annual scientific production and polynomial curve fitting of publication growth in the field; (**b**) average citations per year.

A total of 3304 documents, mainly articles (*n* = 2083, 63.0%) and reviews (*n* = 813, 24.6%), were collected from 1995 to 2022. The average annual growth rate of the literature was 26.7%, peaking in the last decade (2013–2022) in 2014 (+84.5%) and maintaining a positive trend until 2022—the most productive year (*n* = 598, 18.1% of the total). Based on the fitted curve, approximately 706 documents on oligometastatic disease are expected to be published in 2023 (Figure 1a).

The first and only article from 1995 received a total of 1612 citations (mean: 56 citation/year), while considering the 1996–2022 time span, total citations resulted in an average of 35 per year. The year 2010 revealed a peak of 92 citations/document and 4.5 citations/year on average (Figure 1b). 

**Figure 2:**
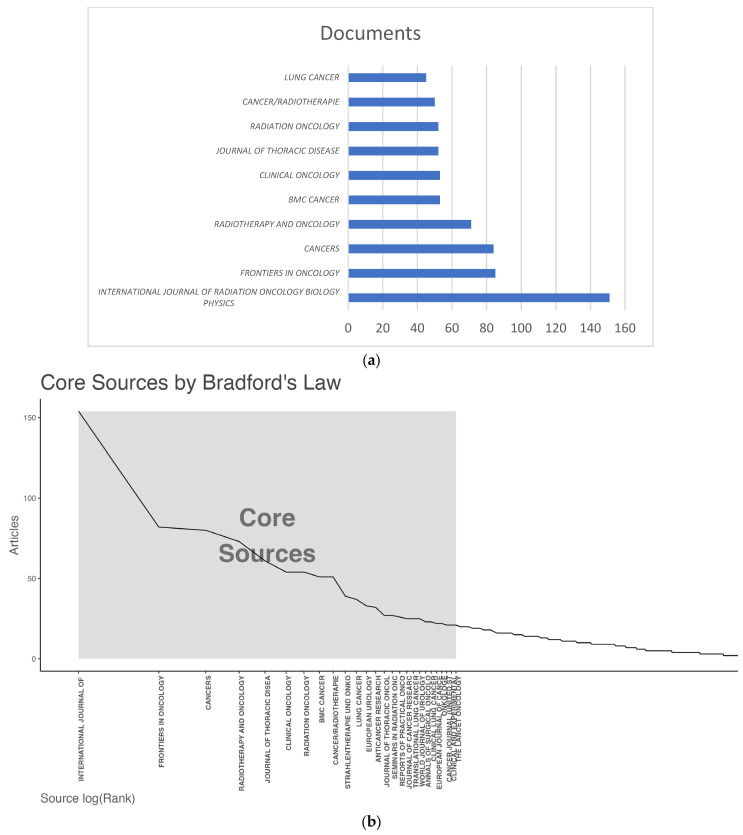
(**a**) Top 10 relevant sources; (**b**) source clustering through Bradford’s law.

Documents were retrieved from 690 sources. The *International Journal of Radiation Oncology Biology Physics* contributed with 151 documents (2003–2022), followed by *Frontiers in Oncology* and *Cancers*, with 85 (2012–2022) and 84 (2018–2022) documents, respectively (Figure 2a). Thirty journals (Figure 2b) constituted the core sources, with a total of 1219 documents (37% of all published documents). Documents from the *International Journal of Radiation Oncology Biology Physics* were the most cited, with a total of 4285 citations, followed by documents from *Radiotherapy and Oncology*, with 2053 total citations. 

**Figure 3:**
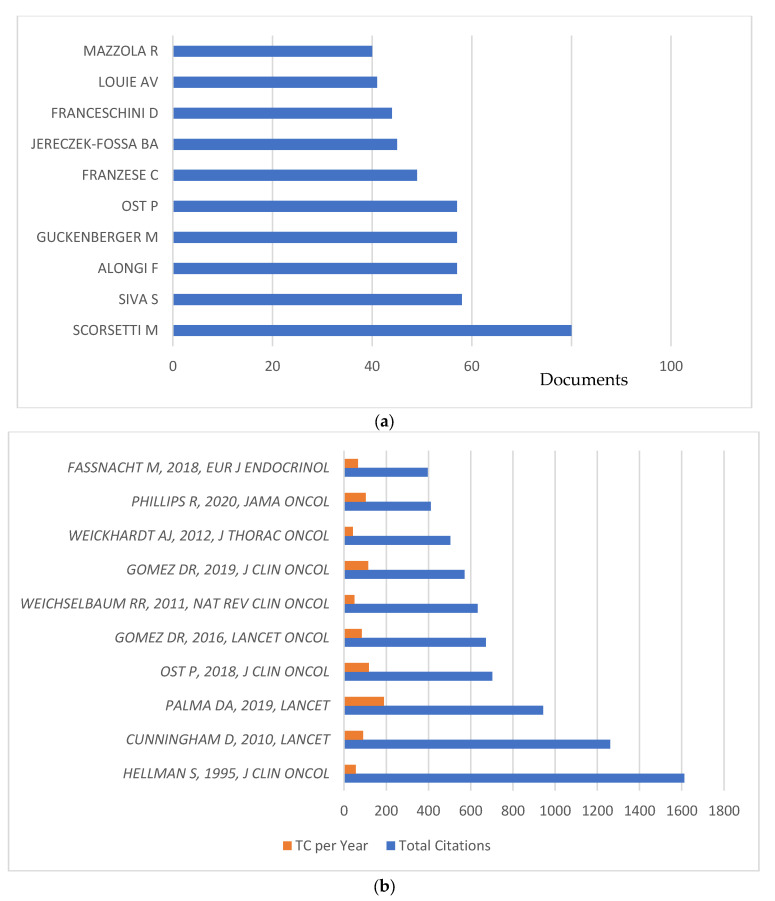
(**a**) Top 10 relevant authors; (**b**) top 10 cited documents.

Overall, 15,176 authors published on the topic, with an average of eight authors/publication. The top 10 authors are shown in Figure 3a. The top two most relevant contributors were Marta Scorsetti and Shankar Siva, with 80 and 58 publications, respectively, followed by Filippo Alongi, Matthias Guckenberger, and Piet Ost, with 57 documents each. The analyses revealed that the majority of contributors (10,902 authors, 71.8%) were occasional authors who contributed to one document. Total citations and average citations/year can be seen Figure 3b for the top 10 cited documents. As mentioned above, the document from Hellman S et al. (*J. Clin. Oncol*., 1995) received the highest number of citations (1612), followed by Cunningham D et al. (*Lancet*, 2010) with 1261 citations (average 90 citations/year) and Palma DA et al. (*Lancet*, 2019) with 943 citations (average 189/year). Among all considered documents, 18.1% were publications with authors from at least two countries. 

**Figure 4:**
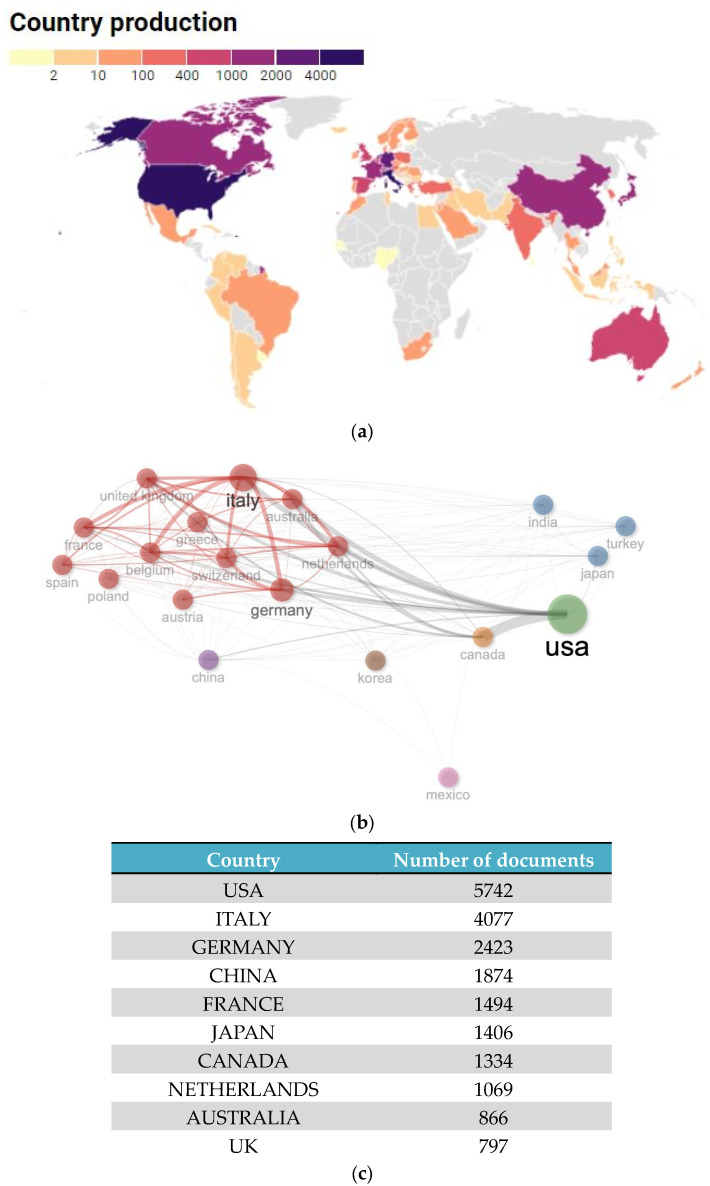
(**a**) Country-specific production; (**b**) top 10 most productive countries; (**c**) collaboration network among countries. Each vertex represents an item, and its size is proportional to the item’s occurrence. Each cluster can be considered as a topical macro-area, and the colors represent the cluster to which each word belongs.

From 1995, 69 countries contributed to the literature (Figure 4a,b); the USA and Italy were the top contributors, with 5472 and 4077 documents, respectively. Documents from the USA and Italy were also the most cited (Figure S1), with a total of 18,010 and 4901 citations, respectively.

Figure 4c shows the relationship network among countries. Each vertex represents an item, and its size is proportional to the item’s occurrence. Each cluster can be considered as a topical macro-area, and the colors represent the cluster to which each word belongs. The USA had a strong connection with Canada, Germany, and Italy, and there was a clear tendency of European countries (red) to cooperate. Mexico, China, and Korea constituted a node of their own, as did India, Japan, and Turkey. The USA, Italy, and Germany were the countries with the greatest numbers of multiple-country documents (i.e., documents with authors from at least two countries; Figure S2), with multi-country publication ratios of 0.13, 0.18, and 0.21, respectively. 

The top three institutions in terms of publication volume were the University of Texas MD Anderson Cancer Center, Houston, TX (*n* = 309 documents), the Memorial Sloan Kettering Cancer Center, New York, NY (*n* = 302), and the University of Toronto, Toronto, ON (*n* = 276) (Figure S3).

### 3.2. Themes and Trends

**Figure 5:**
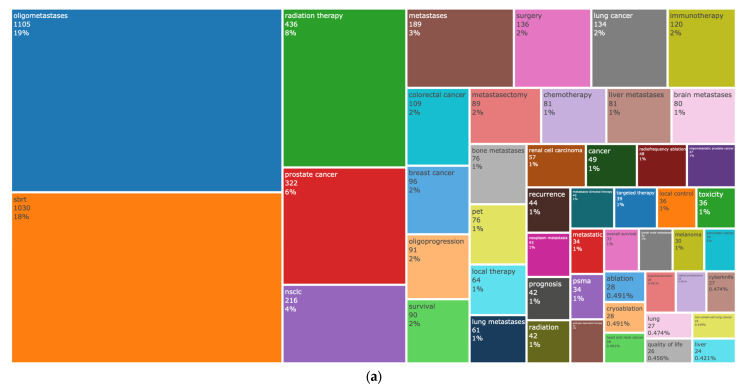

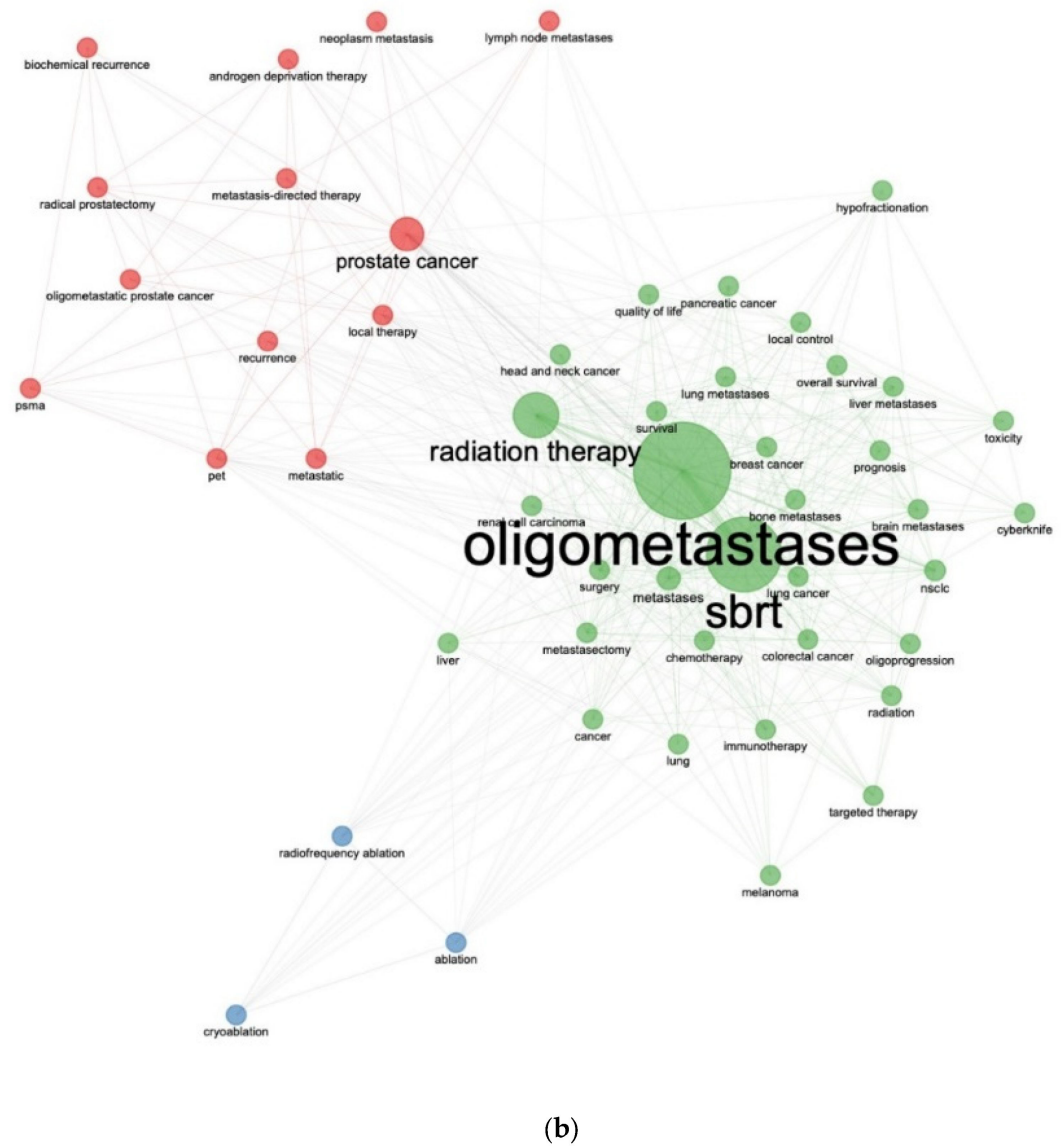
(**a**) Tree map of keywords’ occurrence; (**b**) keyword network analysis.

A total of 3576 different keywords were retrieved from the documents. Analysis of keywords’ occurrence is provided in Figure 5a. Among all keywords used by authors, the top three were *oligometastases* (19%), *SBRT* (18%), and *radiation therapy* (8%), indicating that RT techniques play a very prominent role in the oligometastatic setting. The network among keywords (Figure 5b) showed three different clusters: (i) the main *oligometastases* cluster (green), with a close relationship with *SBRT* and *radiation Therapy*; (ii) the *prostate cancer* cluster (red), a research hotspot in the field able to constitute a line of research on its own; and (iii) a cluster comprising radiofrequency ablation techniques. 

As shown in Figure S4, the change in trend themes over the period 2009–2022 demonstrated the evolution of hotspots in oligometastasis research; themes regarding “locoregional treatment”, “organ motion”, and “immunotherapy” are the most recent trend topics, mainly developed from 2019 to 2022, while “high-dose chemotherapy”, “whole-brain radiotherapy”, and “metastatic breast cancer” saw their main development during 2009–2018. In the recent literature, prostate cancer is the most presented histology, followed by breast cancer and non-small-cell lung cancer. 

The thematic map (Figure S5) confirmed *SBRT*, *radiation therapy*, and *recurrence* as basic topics (i.e., higher values of centrality and lower values of density). *MR-linac* is a topic that has gained attention; however, it is settling into niche topics, given its low availability. *Immunotherapy* and *targeted therapies* are emerging topics that are consolidating as key issues in this context. *Radiofrequency ablation*, *cryoablation*, and *microwave ablation* are confirmed as isolated topics. 

**Figure 6:**
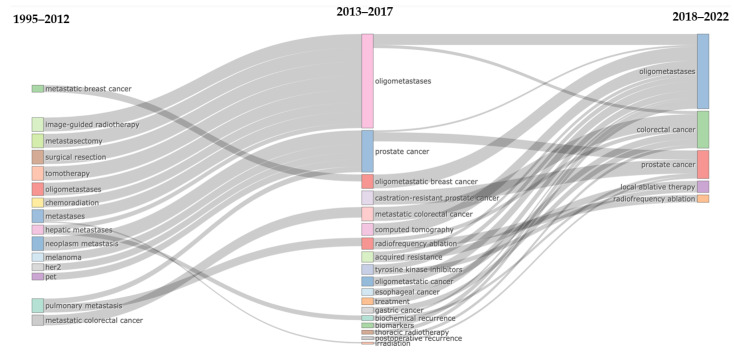
Thematic evolution of keywords.

Figure 6 shows the evolution of the main thematic areas and their relationships during the considered periods (1995–2012, 2013–2017, and 2018–2022). In 1995–2012, several niches of research were present. Among them, *image-guided RT*, *metastasectomy*, *surgical resection*, *tomotherapy*, *chemoradiation*, and *metastases* merged into the *oligometastases* topic in 2013–2017. Several additional niches appeared in the 2013–2017 period, and similarly, a few among them merged into the *oligometastases* topic. *Prostate cancer*, which appeared in 2013–2017 as a strong field of research, continued to draw attention in the following years and, indeed, was still present as one of the main themes in 2018–2022. *Colorectal cancer* was present in all considered periods. 

**Figure 7:**
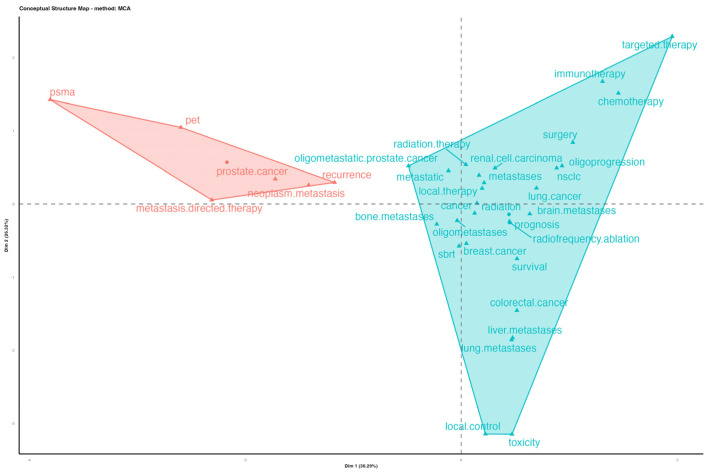
Conceptual structure map: factorial and multiple correspondence analysis of the 50 highest-frequency keywords.

A conceptual structure map was constructed through a clustering and multiple correspondence analysis of the 50 author keywords with the highest frequency (Figure 7). This map allows for the representation of high-dimensional data through a low-dimensional space and a two-dimensional graph. The distance between points on the plane illustrates the similarity between the keywords; meanwhile, when a keyword approaches the center point on the graph, it indicates its centrality in receiving significant attention over the years [23]. Two groups have been identified: one involving the role of radiation oncology as main actor, further consolidating its pivotal role in the treatment of oligometastases, and another with prostate cancer as the main keyword. The latter, through its linked keywords (i.e., PET, PSMA, recurrence, metastasis-directed therapy), emphasizes the conveyed value as a consolidated field of research in the area of oligometastases. 

## 4. Discussion

The present bibliometric analysis included 3304 documents published between 1995 and 2022, mainly consisting of original articles (*n* = 2083, 63.0%) and reviews (*n* = 813) from 69 different countries. 

The number of articles in the field of oligometastatic cancer has increased significantly every year (average annual literature growth rate of 26.7%), indicating a growing interest in the subject, starting from the first paper published in 1995, where the definition was proposed for the first time. Indeed, the publication authored by Hellman S et al. [18] received the highest number of citations (*n* = 1612), paving the way for a new era in metastatic patients. 

With regards to countries and geographical distribution, the United States leads the field, with the highest number of publications, followed by Italy and Germany. The three most productive academic institutions are located in the US, while the fourth is in Canada, mirroring the affiliation of one of the most highly cited authors in the field, David Palma. 

An analysis of the top 19 most productive countries provides further insight into these data. The results showed the most relevant cluster of collaboration occurring between the USA and Canada, followed by two smaller clusters involving Italy and Germany as the most productive players among the European countries. 

To determine the nature and interest of the articles that have had a major impact on the scientific literature in the field, an analysis of the most cited documents was performed. The first and most highly cited article is considered to be a milestone in oligometastatic research and accounts for 1612 citations in total, i.e., 2.8% of the total number of overall citations of the papers included in our analysis. Written in 1995 by Hellman S et al. [18], the article introduces a new paradigm of metastatic disease, challenging the conventional understanding and ushering in what can be defined as the oligometastatic era. This era is constantly showing that patients with a limited burden of metastatic disease can potentially achieve a longer progression-free survival (PFS) and overall survival (OS) with appropriate MDT, such as metastasectomy and SBRT. The latter is the result of the advances in radiation therapy (RT) technology over the years, making it a safe choice to ensure the postposition of burdensome systemic treatment, leading to an improvement in the quality of life (QoL) of the patients, resulting in a better ratio of time on–time off chemotherapy or hormone therapy.

Almost all of the published documents were edited by journals mostly covering the field of radiation oncology. Interestingly, of the top 10 most cited publications, 8 are radiotherapy-related, and all of the most cited authors are recognized radiation oncologists. It is noteworthy to highlight that the research strategy did not include keywords such as “radiation therapy” or “SBRT”, underscoring the significant impact that radiotherapy covers in the field of oligometastatic disease/states. 

Among the 10 most cited articles, the definition of “oligometastatic disease” is strictly associated with the role of RT, and particularly with the use of SBRT in this setting. From the second most cited publication onwards, the main topics covered are related to the clinical evidence supporting the use of SBRT and its ablative role in the oligo scenario. SBRT has been shown to be highly effective in achieving local control without increasing toxicity. The available evidence-based data for the use of MDTs, as shown in the majority of the papers, were explored principally in lung (3), prostate (3), and colorectal cancers (1). 

Two clinical randomized trials occupy the third and fourth position; the former [24] constitutes one of the solid bases in the treatment of oligo patients from different histologies (mainly breast, lung, and prostate), “SABR-COMET”, and the latter represents one of the most important trials in the prostate cancer oligometastatic setting, “STOMP” [16]. Recent published updated follow-up data from SABR-COMET confirmed a greater and sustained advantage in OS over time in patients treated with MDT, and with no detrimental impact on QoL. 

The studies conducted by Gomez et al. [25,26] on oligometastatic lung cancer appear in the fifth and seventh positions, with the same population but a longer follow-up in the 2019 version [26]. The trial, a multi-institutional phase II randomized study, reported that local consolidative therapy (LCT) with RT or surgery improved PFS and delayed new disease in patients with oligometastatic non-small-cell lung cancer (NSCLC) that did not progress after front-line systemic therapy. 

After that, the study published by Weickhardt et al. in 2012 [27] was a retrospective analysis of patients with metastatic NSCLC harboring ALK+ and EGFR-MT mutations and with oligoprogressive disease (i.e., progression within the central nervous system and/or limited systemic sites) while receiving crizotinib or erlotinib. Local ablative therapy (LAT) was combined with the continuation of the same targeted therapy. The study found that this approach was associated with improved PFS and OS compared to changing therapy or continuing systemic therapy alone. 

Scrolling through the rankings, almost at the end, we found the “ORIOLE” study by Phillips et al. [28], published in 2020—a phase II randomized clinical trial in which the role of ablative SBRT was explored in oligometastatic prostate cancer patients. The study utilized a 2:1 randomization ratio between patients receiving SABR or undergoing observation. The primary endpoint was the assessment of progression at 6 months, based on prostate-specific antigen level increase, conventional imaging, symptomatic progression, ADT initiation for any reason, or death. A total of 54 patients with recurrent hormone-sensitive prostate cancer were enrolled and treated with SBRT, demonstrating that RT is a safe and effective modality for MDT, resulting in improved PFS compared to observation.

These findings highlight the importance of prospective randomized clinical trials investigating the oligometastatic state and integrating advanced imaging techniques and biological markers to better characterize this transitional disease state. Such research would further our comprehension of the biology behind oligometastasis and contribute to the creation of more tailored and successful treatment plans for individuals with limited metastatic disease.

This is also the topic explored in the review proposed by Weichselbaum and Hellman [29], titled “Oligometastases revisited”, which restates the concept that the oligometastatic state is a distinct clinical entity. This emphasizes the importance of determining molecular markers in order to distinguish oligometastatic from polymetastatic disease, together with the integration of targeted treatments with MDT. 

The possibility of combining MDT with other techniques—not just SBRT—is underlined by the fact that one of the most cited works (the second in order of appearance), by Cunningham et al. [30], concerns the treatment of liver and lung metastases from colorectal cancer with radiofrequency ablation. Surgical resection of the liver and lung metastases represents the standard of care for resectable oligometastatic disease and, when it is possible to perform, improves 5-year and 10-year survival rates. Improvement of nonsurgical strategies for unresectable lesions has increased the number of patients undergoing complete local treatment of liver metastases with radiofrequency ablation.

The results of the machine-learning-based cluster analysis of the top 50 author keywords provide valuable insights into the semantic cohesion within the domain of oligometastatic cancer research. The three main groupings of co-occurring words identified in this study highlight the key areas of focus within the field and the interconnections between them.

The first cluster, with “oligometastases” as the main contributor, indicates that this topic has emerged as a dominant area of research and has mainly focused on recent treatment modalities, such as RT and SBRT. The tight nest of this cluster with the main big killers, such as “breast cancer” and “colorectal cancer”, and the main sites of oligometastases, such as “liver metastases”, “bone metastases”, and “brain metastases”, suggests the research interest in developing targeted treatments for these types of cancer and their associated metastases.

The second cluster, centered on the term “prostate cancer”, indicates that this disease is an important area of focus within the topic of oligometastases. The use of “positron emission tomography (PET)”, “prostate-specific membrane antigen (PSMA)”, “androgen deprivation therapy”, “biochemical recurrence”, “radical prostatectomy”, and “lymph node metastases” within this cluster highlights the specific challenges and opportunities within this area of research.

The third cluster, composed of terms such as “radiofrequency ablation”, “ablation”, and “cryoablation”, suggests that researchers are exploring minimally invasive treatment methodologies for metastatic cancer. Radiofrequency ablation and cryoablation are two examples of minimally invasive techniques that can be used to destroy cancerous cells. This cluster indicates that researchers are exploring the use of these techniques to treat metastatic cancer, although further research is needed to evaluate their efficacy and safety.

Overall, the results of this analysis highlight the diverse and interconnected nature of metastatic cancer research. The clustering of keywords provides insights into the key areas of focus within the field, and the interconnections between these areas suggest that researchers are exploring multiple avenues to develop more effective treatments for metastatic cancer.

Perchance owing to the nascent nature of the topic at hand, multiple keywords have been employed in manifold occurrences as acronyms or in their nominal or adjectival forms, evincing a profound splintering of the keyword landscape. The current fragmented scenario has been ameliorated by means of an apt synonymic analysis; nevertheless, additional integration may be necessary to refine the exploratory tactics for further studies relying on the review methodology.

Analyzing the theme development and interest, overall, the observed trends highlight the dynamic and ever-changing nature of research in this domain. As new research areas emerge and existing ones converge, it is crucial for researchers to stay attuned to these developments and adapt their approaches accordingly. The continued importance of oligometastases as a focal point of research underscores the significance of exploring new treatment modalities for metastatic cancer, while the persistent interest in prostate and colorectal cancers highlights the need for ongoing investigation in these areas.

Notably, the link between radiotherapy and topics of side-fields like *immunotherapy* and *targeted therapies* represents an opportunity for further studies in this setting, dealing with the combination of these treatment strategies.

The present work has certain shortcomings; the dataset was gathered from just one electronic source (Scopus) that could have potentially influenced our findings, and the quantity of citations fluctuates over time, so this portion of the study should be viewed as tentative and subject to change in the long run. Finally, self-citations may be considered a source of potential bias for the study in general.

While the abovementioned limitations may have a minor impact on the overall results, they are unlikely to alter the main trends shown in the work.

## 5. Conclusions

To conclude, the present bibliometric study represents a detailed summary of the most influential studies dealing with the oligometastatic state. This analysis highlights the interests and future developments in this field and can serve as a benchmark to identify the area for the attention of researchers worldwide and contribute to the increasing scientific work in this rapidly evolving area.

## Data Availability

Data are available upon reasonable request to the corresponding author.

## Supplementary Materials

The following supporting information can be downloaded at: https://www.mdpi.com/article/10.3390/cancers15153902/s1, Figure S1: Top 10 most cited countries and number of citations; Figure S2: Top 10 most productive countries; Figure S3: Top 10 most relevant affiliations and number of documents; Figure S4: Evolution of keywords in the time span 2009–2021; Figure S5: Thematic map.
