# Supplementary material for: A Bibliometric Analysis of the Oligometastatic State over the Last Two Decades: A Shifting Paradigm for Oncology? An AIRO Oligometastatic Study Group
Source: Cancers (Basel). 2023 Jul 31;15(15):3902. doi: 10.3390/cancers15153902 (PMC10417037; doi:10.3390/cancers15153902)
Supplement: Supplementary file 1 [file cancers-15-03902-s001.zip › cancers-2500331-supplementary.pdf]

Supplementary Materials: A Bibliometric Analysis of the Oligometastatic State over the Last Two Decades: A Shifting Paradigm for Oncology? An AIRO Oligometastatic Study Group

Giulia Marvaso, Federico Mastroleo, Giulia Corrao, Mattia Zaffaroni, Maria Giulia Vincini, Paolo Borghetti, Francesco Cuccia, Manuela Federico, Giampaolo Montesi, Antonio Pontoriero, Davide Franceschini, Ciro Franzese, Marta Scorsetti and Barbara Alicja Jereczek-Fossa

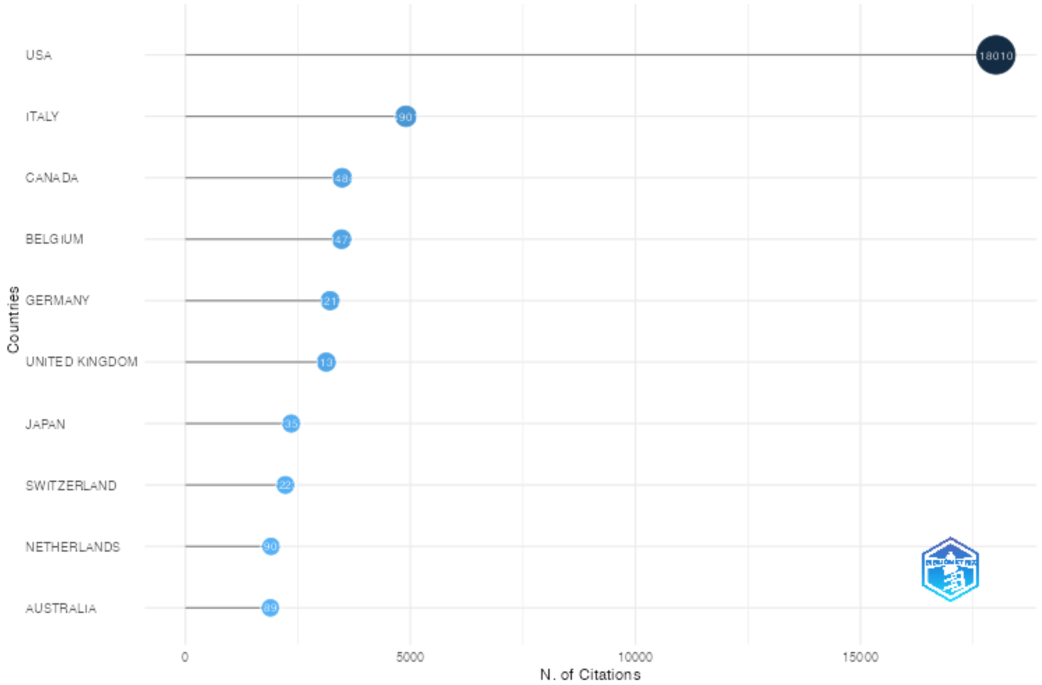

Figure S1. Top ten cited countries and number of citations.

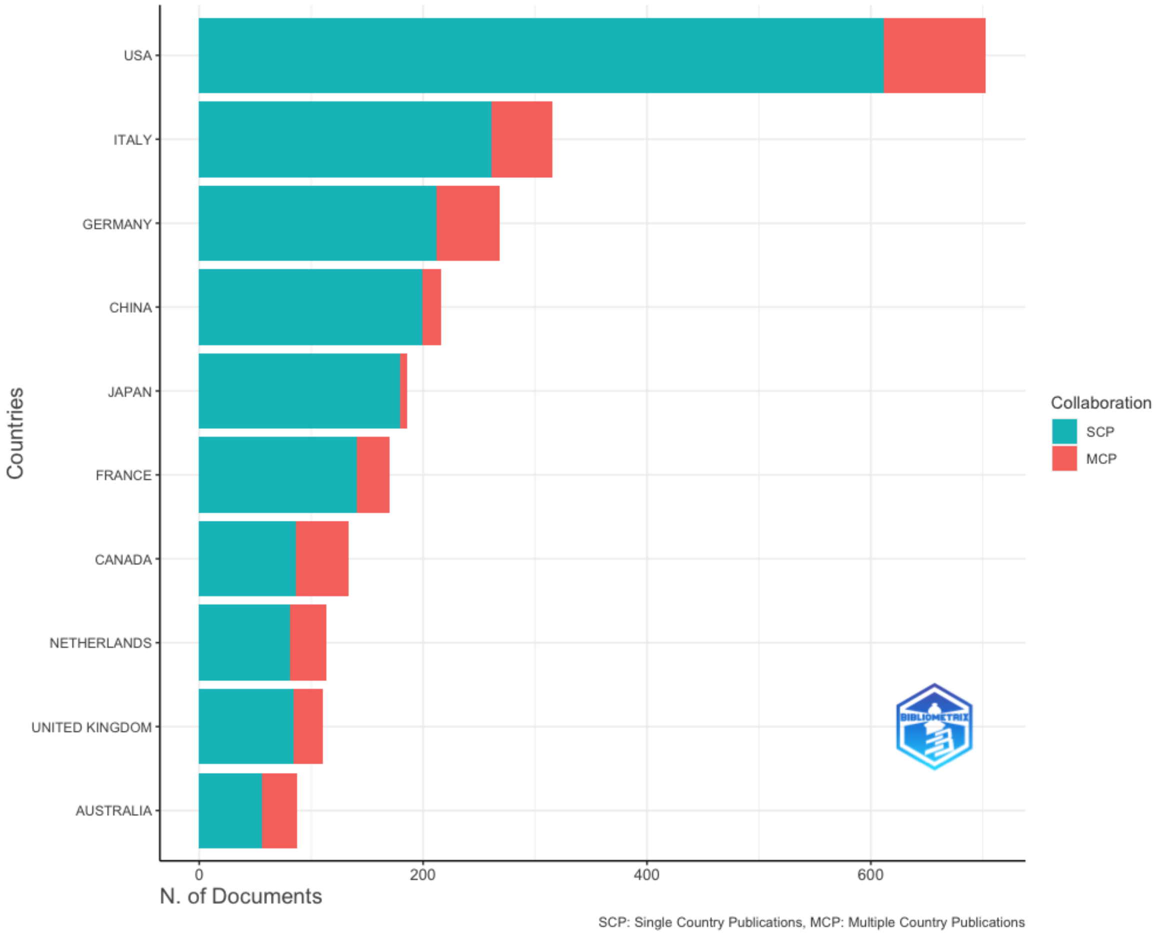

Figure S2. Top ten most productive countries.

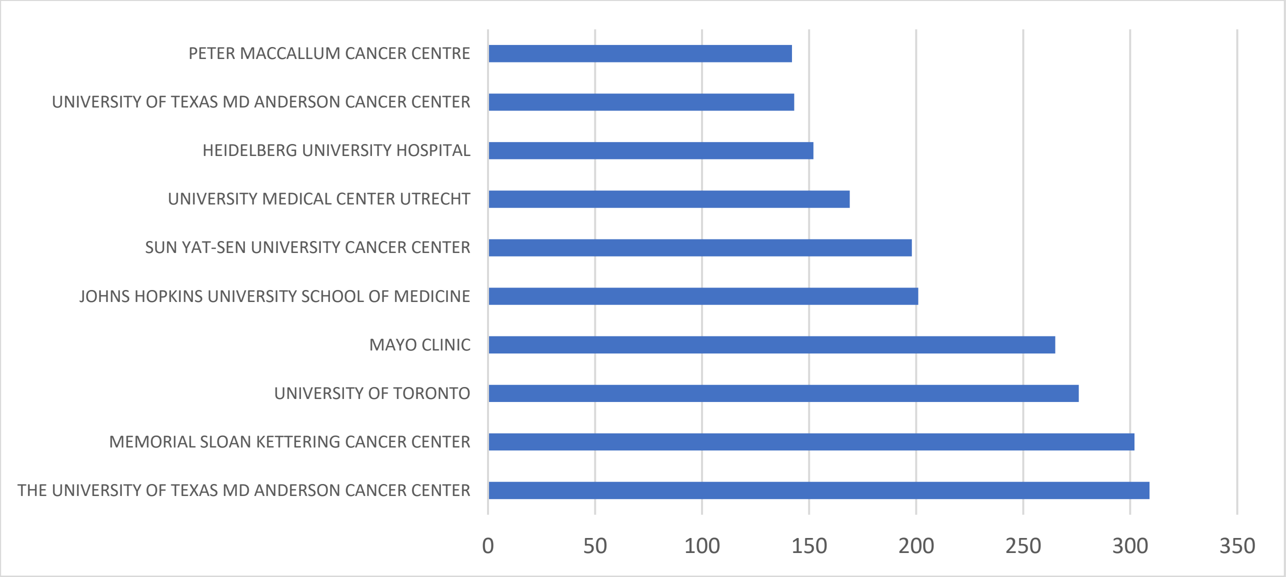

Figure S3. Top ten relevant affiliations and number of documents.

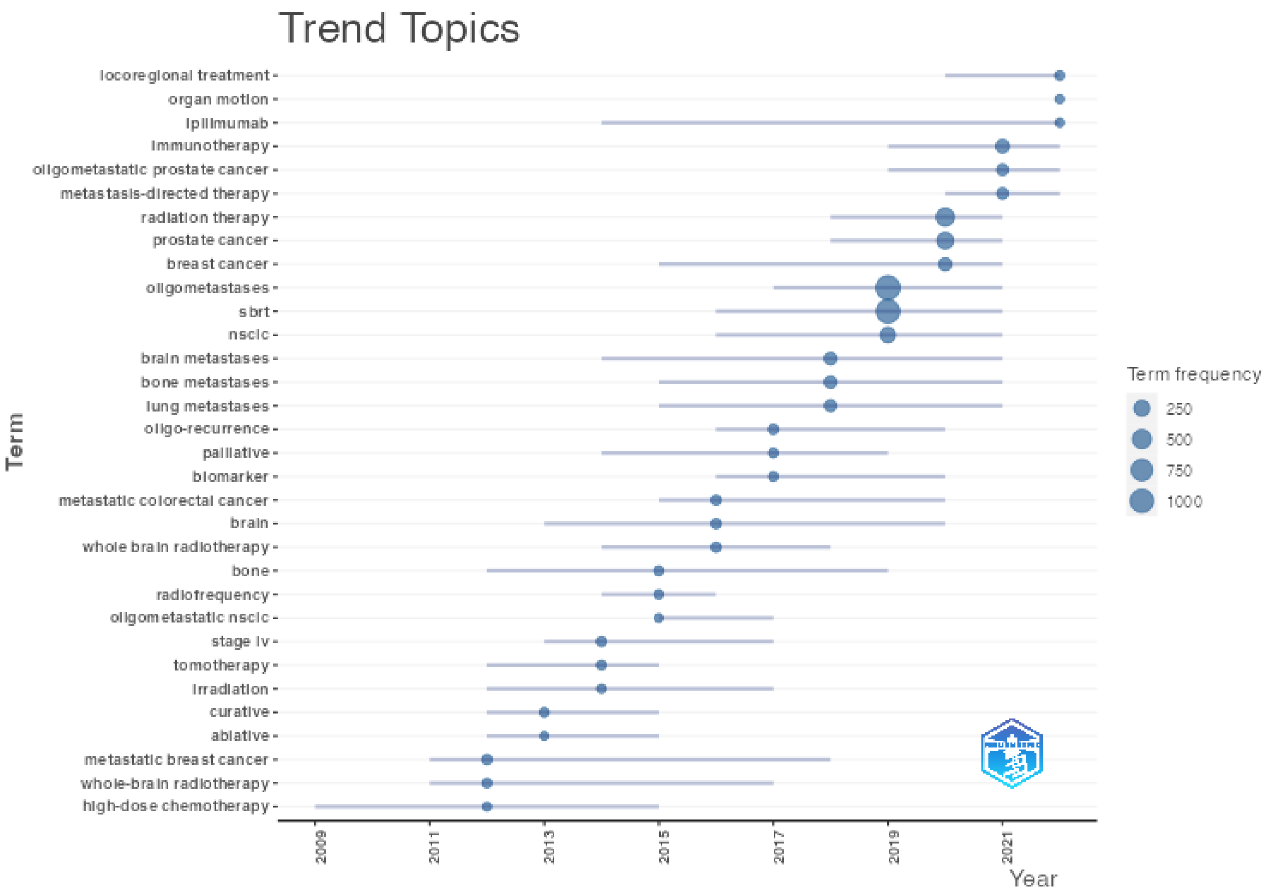

Figure S4. Evolution of keywords in the time-span 2009-2021.

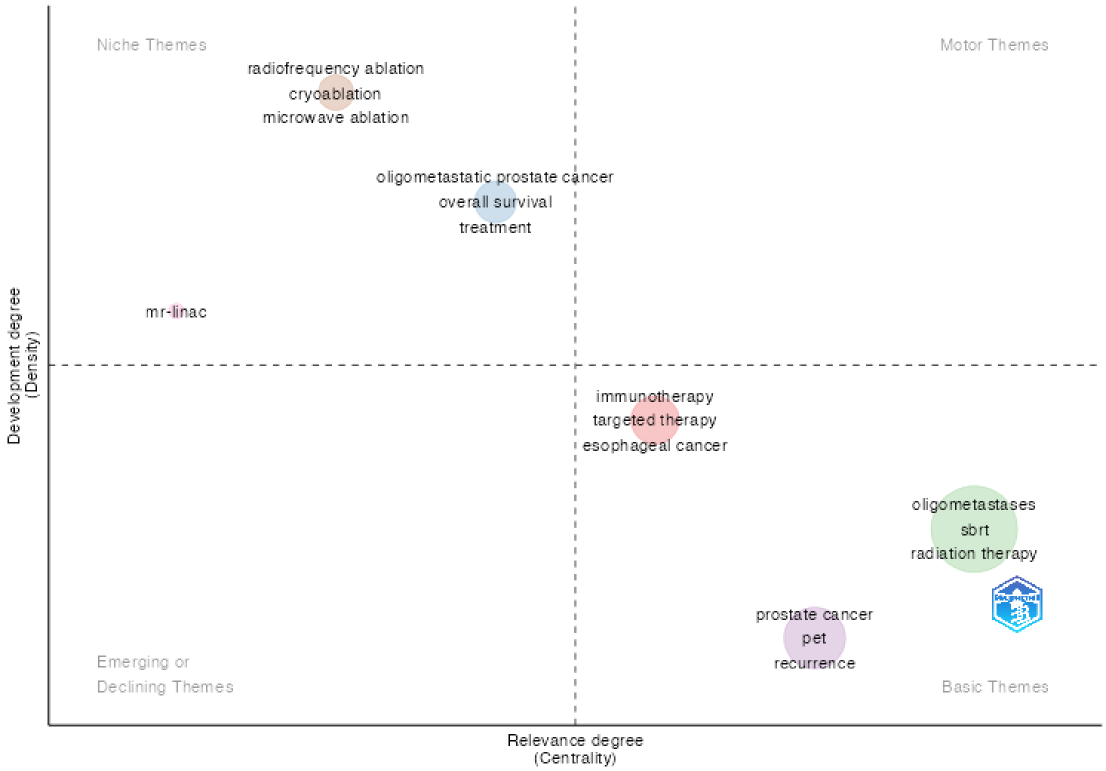

Figure S5. Thematic map.
